# Disease-specific survival for limited-stage small-cell lung cancer affected by statistical method of assessment

**DOI:** 10.1186/1471-2407-7-31

**Published:** 2007-02-20

**Authors:** Patricia Tai, Judith-Anne W Chapman, Edward Yu, Dennie Jones, Changhong Yu, Fei Yuan, Lee Sang-Joon

**Affiliations:** 1 University of Saskatchewan, Faculty of Medicine, Saskatoon; Department of Radiation Oncology, Regina, Saskatchewan, Canada; 2 National Cancer Institute of Canada Clinical Trials Group, Queen's University, Kingston, Canada; 3 Division of Radiation Oncology, Department of Oncology, University of Western Ontario, London, Ontario, Canada; 4 University of New Mexico, Division of Hematology/Oncology, Cancer Research and Treatment Center, Albuquerque, New Mexico, USA; 5 University of New Mexico, Department of Internal Medicine, Division of Epidemiology and Biostatistics, Albuquerque, New Mexico, USA

## Abstract

**Background:**

In general, prognosis and impact of prognostic/predictive factors are assessed with Kaplan-Meier plots and/or the Cox proportional hazard model. There might be substantive differences from the results using these models for the same patients, if different statistical methods were used, for example, Boag log-normal (cure-rate model), or log-normal survival analysis.

**Methods:**

Cohort of 244 limited-stage small-cell lung cancer patients, were accrued between 1981 and 1998, and followed to the end of 2005. The endpoint was death with or from lung cancer, for disease-specific survival (DSS). DSS at 1-, 3- and 5-years, with 95% confidence limits, are reported for all patients using the Boag, Kaplan-Meier, Cox, and log-normal survival analysis methods. Factors with significant effects on DSS were identified with step-wise forward multivariate Cox and log-normal survival analyses. Then, DSS was ascertained for patients with specific characteristics defined by these factors.

**Results:**

The median follow-up of those alive was 9.5 years. The lack of events after 1966 days precluded comparison after 5 years. DSS assessed by the four methods in the full cohort differed by 0–2% at 1 year, 0–12% at 3 years, and 0–1% at 5 years. Log-normal survival analysis indicated DSS of 38% at 3 years, 10–12% higher than with other methods; univariate 95% confidence limits were non-overlapping. Surgical resection, hemoglobin level, lymph node involvement, and superior vena cava (SVC) obstruction significantly impacted DSS. DSS assessed by the Cox and log-normal survival analysis methods for four clinical risk groups differed by 1–6% at 1 year, 15–26% at 3 years, and 0–12% at 5 years; multivariate 95% confidence limits were overlapping in all instances.

**Conclusion:**

Surgical resection, hemoglobin level, lymph node involvement, and superior vena cava (SVC) obstruction all significantly impacted DSS. Apparent DSS for patients was influenced by the statistical methods of assessment. This would be clinically relevant in the development or improvement of clinical management strategies.

## Background

The Cox proportional hazards model [1] has been the standard tool for multivariate assessments of the influence of prognostic/predictive factors for censored survival data. It may encounter serious difficulties with departures from the proportional hazards assumption, even when the departures are not readily detected by commonly used statistical analytic methods [2].

As well, it has been questioned whether a standard survival analysis framework is appropriate if there is an identifiable mixture of "cured" and "non-cured" patients [3-5], for whom there may be distinguishably different factor effects [4,6]. Various models have been used in the context of censored patient data to assess the cured fraction, e.g. Boag log-normal with a cure-fraction parameter [7], modified-Boag model with covariates [6], Cox model for grouped survival data [3], logistic mixture model [4], or a Box-Cox transformation on the population survival function that includes both the mixture cure model and the biologic promotion time cure model [5]. Generalizability of survival analysis may be accomplished with flexible models that comprehensively span a range of options, such as the transformation cure model [5], or the Pettit model [8] which encompasses both Cox and Generalized F models. However, increases in model complexity may affect study conclusions, leading to non-identifiability or instability of results [4,5,9]. Higher level statistical (research) assessment is operationally essential for complex models.

Meanwhile, there is a clinical practice movement to the use of real-time medical decision making tools like Adjuvant! Online [10]. Users input prognostic/predictive factor data, and are quickly provided with estimated survival, without the need to consult a statistician or consider underlying modeling, which is provided within the software. Modeling is operationally becoming a new de facto staging system, which is iteratively updated by a provider with accumulating literature-based evidence.

Our goal here was to compare disease-specific survival (DSS) obtained by modeling of prognostic/predictive effects for a few simple model systems, to set the stage for a re-examination of commonly available clinical factors for limited-stage small-cell lung cancer, where there is a need to develop a new staging system. Standard Kaplan-Meier and Cox modeling were used because of their prevalence in clinical practice, along with two different types of log-normal models, the Boag cure-rate [7] and log-normal survival analysis [11].

Boag demonstrated more than five decades ago that a log-normal model was appropriate for breast cancer patients [7]. Royston [11] found that the prognosis for breast cancer patients differed by up to a year, depending on whether one utilized a Cox or log-normal assessment. Tai et al. validated the Boag log-normal model for the estimation of survival in patients with limited-stage small-cell lung cancer (SCLC) [12]. Overduin used follow-up to events in Tai's data to examine the goodness of fit for the Weibull, gamma, and lognormal models [13]. The fit was poor for both the Weibull and gamma (p < 0.001, in each instance), while acceptable for the log-normal (p = 0.37). Tai's updated long-term Saskatchewan patient cohort data were used here for the model comparisons. We hypothesized that there might be substantive differences in DSS for the same limited-stage SCLC patients, if Boag log-normal (cure-rate model) [7], Kaplan-Meier [14], Cox [1], and log-normal survival analysis [15,16] were used.

## Methods

### Patients

Between 1981 and 1998, 1417 cases of SCLC were diagnosed in Saskatchewan, Canada and entered in the Saskatchewan cancer electronic registry prospectively. Of these, 244 had limited stage disease and were treated with chemotherapy and thoracic radiotherapy delivered with curative intent, with or without prophylactic cranial irradiation [12]. For the whole series, only six patients did not have any chemotherapy. Cisplatin-containing regimen was given to 54 patients. The remaining patients had non-cisplatin-containing regimens.

To facilitate the comparison of the different fractionation schemes used for radiotherapy, we calculated the biologically effective dose (BED) [17,18], using the linear-quadratic model:

BED=nd×[1+dα/β]
 MathType@MTEF@5@5@+=feaafiart1ev1aaatCvAUfKttLearuWrP9MDH5MBPbIqV92AaeXatLxBI9gBaebbnrfifHhDYfgasaacH8akY=wiFfYdH8Gipec8Eeeu0xXdbba9frFj0=OqFfea0dXdd9vqai=hGuQ8kuc9pgc9s8qqaq=dirpe0xb9q8qiLsFr0=vr0=vr0dc8meaabaqaciaacaGaaeqabaqabeGadaaakeaacqWGcbGqcqWGfbqrcqWGebarcqGH9aqpcqWGUbGBcqWGKbazcqGHxdaTdaWadaqaaiabigdaXiabgUcaRmaalaaabaGaemizaqgabaacciGae8xSdeMaei4la8Iae8NSdigaaaGaay5waiaaw2faaaaa@3EFD@

The median BED to the chest was 46.9 Gy_10 _(range 22.6–66.1), corresponding to a median dose of 37.5 Gy in 15 fractions within 19 days (range 20 Gy in 15 fractions within 20 days to 60 Gy in 30 fractions within 44 days), where Gy_10 _is the BED when α/β is 10. Chemotherapy regimens and radiotherapy techniques were those utilized by clinicians during the study period. Current patient management may differ; however, the focus of this work is the comparison of DSS assessed by different statistical methods in the same cohort of patients.

More detailed data for the current study were obtained from the chart review for individual patients, followed to the end of 2005 by a health record technician, and checked by an oncologist (PT).

### Statistical Methods

#### Event

Death with or from lung cancer, for DSS, was the event of interest here. The last recorded SCLC death was at 1966 days of follow-up (5.4 years), and the second last event was at 1789 days (4.9 years). The lack of events after 1966 days precluded comparison after 5 years. Follow-up was censored at death from other causes for the Kaplan-Meier, Cox, and log-normal survival analyses. One patient had unknown status at last follow-up, and was excluded from the analyses since the Boag cure-rate model requires alive/dead categorization for each patient, and where appropriate, knowledge of type of death.

### Statistical Modeling

Cox and log-normal survival analyses utilized both censored and uncensored data towards the estimation of a common set of factor effects [19]. For these survival analyses frameworks [19], we considered the values of the survivor functions at a time when few (or no) events are expected as reasonable estimates of the proportion cured, without specifying a cured-rate parameter [19].

### Cox model

The Cox model assumes proportional hazards; this assumption was checked graphically with plots of cumulative hazard against follow-up time [19].

### Log-normal models

Neither the Boag cure-rate nor log-normal survival analysis require the Cox assumption of proportional hazards. Both the Boag (log-normal) cure-rate model and log-normal survival analyses assume that the logarithm of lung cancer survival time has a standard normal distribution; quantiles obtained for times of SCLC cancer deaths were utilized to check this assumption for the 2005 update with a quantile-quantile (Q-Q) plot and a chi-square goodness of fit test against the normal distribution.

#### 1. Boag (log-normal) cure-rate model

Boag (log-normal) cure-rate modeling begins with the classification of patients as being "cured" (C) or "uncured" (1-C) at a particular length of follow-up [7], to define four groups: Group 1 patients died of SCLC; Group 2 died without any SCLC; Group 3 were alive with no sign of SCLC; Group 4 were alive with SCLC cancer present either as local, regional or metastatic disease.

For the proportion who are not cured, 1-C, the survival time T is assumed to be log-normally distributed; Y = ln (T) is normally distributed with mean μ and variance σ^2 ^[7]. Generally, one has to jointly estimate C (>0), μ, and σ at some point in time when the group who will be classified as cured may include patients who are not cured, although they have not yet had an event. Sufficiently long follow-up with a disease like lung cancer will minimize misclassification, since it is well known that few lung cancer patients will recur after 4 or 5 years. C is estimated for the full patient group. The focus of these investigations was the estimation of DSS, as described below.

#### 2. Log-normal survival analysis model

For all patients, the survival time T is log-normally distributed if Y = ln (T) is normally distributed with mean μ (= α + **zβ**), and variance σ^2^, where z are covariate(s). A patient without an event is censored at the last follow-up time for that patient. There is no specific parameter to estimate the proportion cured, but one is not needed since the survivor function provides an estimate of the proportion cured at any point in time, with the estimate improving as follow-up time increases to a period when few (or no) events are expected. The survivor function, S(t), is given by S(t) = 1 - Φ [(ln(t) - μ)/σ], where Φ is the standard normal cumulative distribution.

### Statistical Analyses

Estimates of DSS using the Boag cure-rate model and Kaplan-Meier methods are based on outcomes in defined (sub)groups of patients. Thus, to maintain maximal power here, estimation with the Boag and Kaplan-Meier methods required the full cohort of patients. We reported DSS at 1-, 3- and 5-years and 95% confidence limits for all patients, using the four methods: Boag log-normal, Kaplan-Meier, Cox, and log-normal survival analysis.

The following clinical factors were assessed for effect on DSS in step-wise forward model building with both Cox and log-normal survival analysis: gender, age, site of primary, side of lung cancer, lymphadenopathy, pleural effusion, bronchial obstruction, superior vena cava obstruction, surgical resection, performance status, weight loss greater than 5% in 3 months, and hemoglobin level. Continuous factor values were used where possible, along with full patient follow-up.

Best medical practice in Saskatchewan, under the Canadian National Health system was employed throughout accrual of the patient cohort. In clinical practice, the administration of more aggressive therapy to higher risk patients may mask therapeutic benefit. Changing chemotherapy and radiotherapy management schema and the small size of this cohort precluded investigations by current practice categorizations: type of chemotherapy (platinum vs. non-platinum), use of radiotherapy, radiotherapy dose/schedule, lactic dehydrogenase (LDH) or other lab results. Incomplete or no surgical resection in 230 (94%) of the 244 patients prevented the assignment of TNM stage. We did not systematically collect smoking history nor clinical history about prior malignancies or other co-morbid diseases in the database. However, the extensive clinical follow-up for this cohort was useful for the investigation's focus on survival analyses.

Boag log-normal analysis was performed with an Excel programme [12], a computerization of Boag's original spreadsheet, with some macros that improve efficiency of the iterative maximization; it is available from PT on request. Multivariate regressions and residual checks for log-normal survival analyses were performed with Dynamic 7.0 version of the Biomedical Data Package [20, same as BMDP-XP: program 2L, for log-normal ("accel=lnormal.")]. All other analyses were performed with SAS Version 9.1.3.

Cox and log-normal step-wise forward multivariate regressions involved the addition of a factor if there was a significant likelihood ratio test statistic (p ≤ 0.05 for a χ^2^_(1) _test), and factors are reported here if p ≤ 0.10 in both Cox and log-normal survival analyses. Cox-Snell residual checks were used to assess the final models for both Cox and log-normal survival analyses, and standardized residual checks were made for the log-normal model.

When the same factors were indicated as significantly affecting DSS with both the Cox and log-normal models, categorizations of these factors were used to specify sets of clinical characteristics of interest, for quantitation of DSS by the two model-types. DSS was determined quantitatively at 1-, 3-, and 5- years, and graphically demonstrated with survivor plots across the entire time period.

## Results

The median follow-up of those alive was 9.5 years. One hundred and eighty-four (75.7%) of the 243 patients died from lung cancer: by 1 year, 42 of 238 (17.6%); by 3 years, 173 of 230 (75.2%); and by 5 years, 186 of 223 (83.4%). There was no substantive evidence against the assumption of proportional hazards. The times of lung cancer deaths were reasonably consistent with the log-normal model both in the Q-Q plot and by the chi-squared test (p = 0.42).

Table 1 shows the patient characteristics for this patient cohort. Quantitative estimates of DSS were determined for all patients using the Boag log-normal, Kaplan-Meier, Cox, and log-normal survival analysis, and are listed in Table 2. DSS assessed by the four methods in the full cohort differed by 0–2% at 1 year, 0–12% at 3 years, and 0–1% at 5 years. Log-normal survival analysis indicated DSS of 38% at 3 years, 10–12% higher than with other methods; univariate 95% confidence limits were non-overlapping.

Four factors were found to significantly affect DSS in both multivariate Cox and log-normal survival analyses: surgical resection (p = 0.01, with Cox; p = 0.001, with log-normal), hemoglobin level (p = 0.02, with Cox; p = 0.005, with log-normal), lymph node involvement (p = 0.02, with Cox; p = 0.04, with log-normal), and superior vena cava (SVC) obstruction (p = 0.06, with Cox; p = 0.10, with log-normal). These factors then became the focus for characterizing patients and reporting DSS.

Categorizations of the four significant factors were used to specify four sets of clinical characteristics which were given ad hoc labels according to risk level:

1) Group A, low-risk: complete resection, hemoglobin ≥ 100 for both sexes, no lymph node involvement, no SVC obstruction;

2) Group B, intermediate risk #1: incomplete or no resection, hemoglobin ≥ 100 for both sexes, no lymph node involvement, no SVC obstruction;

3) Group C, intermediate risk #2: incomplete or no resection, hemoglobin < 100 for both sexes, no lymph node involvement, no SVC obstruction;

4) Group D, high-risk: no resection, hemoglobin < 100 for both sexes, lymph node involvement, SVC obstruction.

(Please note: A hemoglobin cut-point of 100 is a medical intervention level for both males and females. It is a value that transfusion is generally recommended at during treatment, irrespective of sex. Also, "incomplete or no resection" for groups B and C includes patients with no surgery, or partial resection, who had residual disease after surgery, and were treated with a combination of chemotherapy and radiotherapy.)

DSS by clinical risk groups, estimated using Cox and log-normal models, is reported in Table 3. DSS assessed by the Cox and log-normal survival analysis methods for 4 clinical risk groups differed by 1–6% at 1 year, 15–26% at 3 years, and 0–12% at 5 years (Table 3). The multivariate 95% confidence limits for the year 1-, 3-, and 5-year estimates of DSS with Cox and log-normal survival models overlap in all instances.

**Figure 1:**
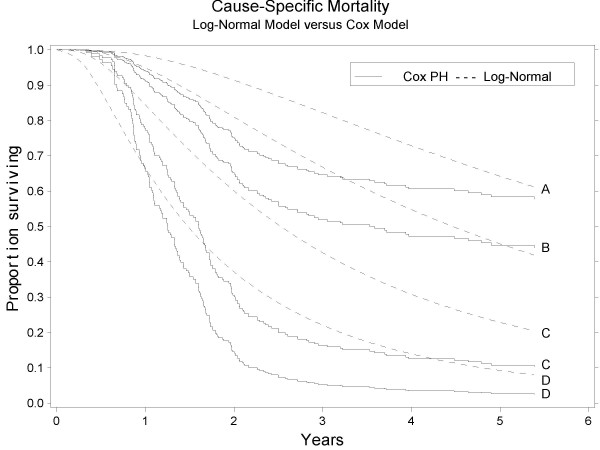
Cause-specific mortality by log-normal and Cox models. 1) Group A, low-risk: complete resection, hemoglobin ≥ 100 for both sexes, no lymph node involvement, no SVC obstruction; 2) Group B, intermediate risk #1: incomplete or no resection, hemoglobin ≥ 100 for both sexes, no lymph node involvement, no SVC obstruction; 3) Group C, intermediate risk #2: incomplete or no resection, hemoglobin < 100 for both sexes, no lymph node involvement, no SVC obstruction; 4) Group D, high-risk: no resection, hemoglobin < 100 for both sexes, lymph node involvement, SVC obstruction.

The log-normal survivor plot (Figure 1) contains a smooth modeling of DSS. The discontinuities observed in the Cox plot (Figure 1) arise because DSS is adjusted at the time of events.

These subgroup effects for the four factors indicated by multivariate analyses, with both Cox and log-normal survival analyses, could not be considered here in a Kaplan-Meier framework. The subgroups contained a total of 8 patients and 4 events.

## Discussion

Patient management decisions are made on the basis of prognosis, or prediction about tumor responsiveness to particular therapeutic regimens. Long-term evidence has supported the relevance of TNM staging or other readily ascertainable clinical characteristics. Kaplan-Meier plots and Cox modeling have been standard assessment tools for decades. Infrequently, other models like the Boag log-normal or log-normal survival analysis have been utilized. SCLC is a disease site for which it might be advantageous to develop a new staging system since the requirement of complete excision to define elements of TNM staging is frequently not met; 94 percent of the patients in this cohort did not have complete resection. We presented here a case study that illustrates differences in DSS obtained using the same patients and the above four methods, each of which has underlying assumptions which may be imperfectly met in any particular data.

Further, SCLC is a particularly good cancer site in which to consider a cure rate framework as very few events are expected after 5 years. The long accrual period (approx. 18 years) may be a limitation; however, the study population is representative of patients treated in this era. With a median follow-up of 9.5 years for these patients, the 5-year DSS estimates should be stable, and the estimates by the four methods differed by only 1%, to be 19 or 20%. This is a survival rate that would be anticipated from the literature.

The survival rates and confidence limits are those which could be reported for the four model-types, although reports usually consider at most 1 or 2 methods. Formal direct statistical tests are not possible between frameworks, e.g. between the semi-parametric Cox and two parametric log-normal models, nor even between Boag log-normal cure rate and log-normal survival analysis. However, 95% confidence limits are provided for DSS obtained by each method. DSS at 3 years was 38% with log-normal survival analysis, 10–12% higher than with other methods. Univariate 95% confidence limits for log-normal survival analysis did not overlap with those obtained for any of the other methods. In the breast cancer setting, Royston [11] observed that prognosis by model-type differed by up to a year depending on whether a Cox or log-normal model was used, and Chapman, et al [16] found up to 8% absolute difference by model-type. Large differences in survival estimates appear possible according to whether one uses a Cox or log-normal model.

The much narrower range in confidence limits for the Boag model reflects that the DSS estimates are for those who have died, while the other three methods incorporate the uncertainty from censored survival times. Further, the Boag model fits an extra parameter, C, for the proportion cured. The estimate of proportion cured, at a time when few or no events are expected, is derived directly with the survivor function for the Cox and log-normal survival analysis models.

The assessments in this cohort for both the Boag and Kaplan-Meier were limited to those with the full patient group due to the size of the SCLC patient cohort which precluded subgrouping with the four factors indicated by multivariate analyses, while more extensive modeling was possible for specific patient characteristics with both Cox and log-normal survival analysis. Apparent differences in DSS of up to 26% at 3-years were seen between these latter two model-types, although all the multivariate 95% confidence limits for the 2 methods overlap. The variability will reflect patient heterogeneity, the imprecise nature of the particular clinical factors investigated, inadequacy of the Cox and log-normal modeling, and sample size. More precise molecular or genomic factors would eventually provide better precision, but could still involve the same assessment methods. Again, large differences between apparent prognosis with the Cox and log-normal survival models have been seen by others: up to a year's difference, by Royston [11]; and up to 8% absolute difference, by Chapman et al. [16]. Absolute differences at 5-years varied less: respectively, 5, 0, 12, and 6%, for patient Groups A-D.

The Cox plot presents discontinuous estimates of DSS, as would the Kaplan-Meier, as DSS is adjusted at events. The Boag model requires the estimate of one more parameter, the cure fraction, which while reasonable in a lung cancer population might not be appropriate for many other cancers with short or medium follow-up. Royston [11] described the log-normal model as a pragmatic tool that provides a continuous estimate. Where the model-type is concordant with the data, smooth log-normal survival analysis may be advantageous over the semi-parametric proportional hazards model. The Cox adjustments at events may unduly alter estimates of DSS at the end of the study period when there are few patients left. In this instance, with few lung cancer deaths expected after 5 years, the DSS estimates should approximate the proportion of patients who are likely 'cured'.

In this study, Groups A to D represent examples of combinations of clinical characteristics for which DSS was determined based on modeling for all patients, rather than subgroup analyses. The main objective of the study was to illustrate the use of different statistical methods to analyze the effects of prognostic/predictive factors on DSS at differing time points. The data used for these investigations accrued from an eighteen year Provinicial cohort of data, recognized externally for its excellence. Unfortuneately, only 244 of the 1417 patients had limited stage disease, and were considered to have received curative management, so they were eligible for these DSS investigations. These investigations are hypothesis generating, requiring external validation in a much larger series. However, follow-up to 5 years was complete for 223/243 (92%) of patients. The reason for considering estimates of 5 year rates was that this would provide the best estimate of the "cured fraction".

Different factors may have been described from additional studies because of the exclusion of factors, small numbers of patients, different patient population, extent of disease and follow-up. Newer factors mentioned in studies include: neuron-specific enolase (NSE) [21], Cyfra21-1 [22], integrin beta1 [23,24], p53 [21], and cytoplasmic MAPK [25].

## Conclusion

In conclusion, among different factors tested, surgical resection, hemoglobin level, lymph node involvement, and SVC obstruction were found to have significant effects on DSS, regardless of model-type. When one attempts to re-evaluate available data aiming towards the formation of a new staging system, different statistical methods are available. Estimation of DSS for patients was influenced by the statistical assessment method. Parametric models should be considered more frequently for survival analysis to assess prognostic and predictive effects; like Royston [11] and Chapman et al. [16], we found here that the log-normal was an appropriate parametric model choice. Multiple methods may be clinically relevant in the development or improvement of clinical management strategies; a software tool might provide estimates with several survival models.

## Competing interests

The author(s) declare that they have no competing interests.

## Authors' contributions

PT, JC: Data analysis and writing of the manuscript;

EY, DJ, SJL: Critical appraisal of the manuscript;

CY, FY: Data analysis.

All authors have read and approved the final manuscript.

## Pre-publication history

The pre-publication history for this paper can be accessed here:



## Figures and Tables

**Table 1:** Patient characteristics (N = 244)

Total number of patients	244
Sex:	
Male	144
Female	100
Age: median	63.72 (range: 38.4–82.0)
Surgery:	
Complete	14
Partial	3
no surgery	227
ECOG Performance Status:	
0	75
1	150
2	11
3	5
4	3
Weight loss:	
> 5%	164
Unknown	21
Hemoglobin:	
Male	128 low, 6 normal, 10 unknown
Female	66 low, 26 normal, 8 unknown
Chemotherapy cycles: median	6 (range: 0 – 27)
Chest Biological Effective Dose: median	46.9 Gy_10 _(range: 22.6–66.1)

Gy_10_, biologically effective doses calculated using the linear-quadratic formula using an α/β = 10 for tumor tissue.

**Table 2:** DSS for full cohort of lung cancer patients (N = 243)

	Estimated disease-specific survival probabilities (in per cent, with 95% confidence intervals, CI) at 1, 3, and 5 years by different methods^1^		
Methods:	1	3	5

Boag	81 (79,83)	28 (26,30)	19 (17,21)
Kaplan-Meier	82 (78,87)	26 (20,31)	19 (14,25)
Cox	82 (77,87)	26 (20,31)	19 (14,25)
Log-normal survival analysis	80 (76,83)	38 (33,43)	20 (17,24)

^1^By 1 year, 42 of 238 (17.6%) of patients died from lung cancer; by 3 years, 173 of 230 (75.2%); and by 5 years, 186 of 223 (83.4%).

**Table 3:** DSS by patient characteristics with Cox and Log normal analysis^1^

Group A – Low risk characteristics: (complete resection, hemoglobin: ≥ 100 for both sexes, no lymph node involvement, no SVC obstruction)			
	Estimated disease-specific survival probabilities (in per cent, with 95% confidence intervals, CI) at 1, 3, and 5 years by different methods		

Methods:	1	3	5

Cox	94 (87,97)	65 (38,82)	58 (31,78)
Log-normal survival analysis	98 (87,99.9)	82 (46,97)	64 (26,92)

			
Group B – Intermediate risk #1: (incomplete or no resection, hemoglobin: ≥ 100 for both sexes, no lymph node involvement, no SVC obstruction)			

	Estimated disease-specific survival probabilities (in per cent, with 95% confidence intervals, CI) at 1, 3, and 5 years by different methods		

Methods:	1	3	5

Cox	91 (85,95)	52 (33,68)	45 (26,62)
Log-normal survival analysis	95 (77,99)	67 (32,91)	45 (16,78)

			
Group C – Intermediate risk #2: (incomplete or no resection, hemoglobin: < 100 for both sexes, no lymph node involvement, no SVC obstruction)			

	Estimated disease-specific survival probabilities (in per cent, with 95% confidence intervals, CI) at 1, 3, and 5 years by different methods		

Methods:	1	3	5

Cox	78 (31,95)	16 (0,67)	11 (0,62)
Log-normal survival analysis	84 (55,97)	42 (14,76)	23 (5,56)

			
Group D – High risk characteristics: (no resection, hemoglobin: < 100 for both sexes, lymph node involvement, SVC obstruction)			

	Estimated disease-specific survival probabilities (in per cent, with 95% confidence intervals, CI) at 1, 3, and 5 years by different methods		

Methods:	1	3	5

Cox	66 (19,90)	5 (0,48)	3 (0, 41)
Log-normal survival analysis	67 (27,93)	22 (4,40)	9 (1,38)

^1 ^The subgroups contained a total of 8 patients and 4 events.

## References

[B1] Cox DR (1975). Partial likelihood. Biometrika.

[B2] Frankel P, Longmate J (2002). Parametric models for accelerated and long-term survival: a comment on proportional hazards. Stat Med.

[B3] Pierce DA, Stewart WH, Kopecky KJ (1979). Distribution-free regression analysis of grouped survival data. Biometrics.

[B4] Farewell VT (1982). The use of mixture models for the analysis of survival data with long-term survivors. Biometrics.

[B5] Yin G, Ibrahim JG (2005). Cure rate models: a unified approach. The Canadian Journal of Statistics.

[B6] Gamel JW, McLean IW (1994). A stable, multivariate extension of the log-normal survival model. Comput Biomed Res.

[B7] Boag JW (1949). Maximum likelihood estimates of the proportion of patients cured by cancer therapy. J R Stat Soc B.

[B8] Ciampi A, Chapman J, Hogg S, Thiffault J (1989). GENCOV: a Fortran program that generates randomly censored survival data with covariates. Computer Methods and Programs in Biomedicine.

[B9] Hallett D, Chapman JW, Gamel JW, McLean IW (1999). Estimation of "cure" rate for intraocular melanoma. Proc Am Assoc Cancer Res.

[B10] Ravdin PM, Siminoff LA, Davis GJ, Mercer MB, Hewlett J, Gerson N, Parker HL (2001). Computer program to assist in making decisions about adjuvant therapy for women with early breast cancer. J Clin Oncol.

[B11] Royston P (2001). The lognormal distribution as a model for survival time in cancer, with an emphasis on prognostic factors. Stat Neerlandica.

[B12] Tai P, Tonita J, Yu E, Skarsgard D (2003). Twenty-year follow-up study of long-term survival of limited-stage small-cell lung cancer and overview of prognostic and treatment factors. Int J Radiation Oncology Biol Phys.

[B13] Overduin S (2004). Use of the lognormal distribution for survival data: inference and robustness. MSc thesis supervised by Stephens MA, Simon Fraser University, Canada.

[B14] Kaplan EL, Meier P (1958). Nonparametric estimation from incomplete observations. J Am Stat Assoc.

[B15] McCready DR, Chapman JA, Hanna WM, Kahn HJ, Murray D, Fish EB, Trudeau ME, Andrulis IL, Lickley HL (2000). Factors affecting distant disease-free survival for primary invasive breast cancer: use of a log-normal survival model. Ann Surg Oncol.

[B16] Chapman JA, Lickley HL, Trudeau ME, Hanna WM, Kahn HJ, Murray D, Sawka CA, Mobbs BG, McCready DR, Pritchard KI (2006). Ascertaining prognosis for breast cancer in node-negative patients with innovative survival analysis. Breast J.

[B17] Brenner DJ, Hall EJ (1992). The radiotherapy workbench.

[B18] Hall EJ, Hall EJ (1994). Time, dose, and fractionation in radiotherapy. Radiobiology for the radiologist.

[B19] Lawless JF (2003). Statistical Models and Methods for Lifetime Data.

[B20] (1993). BMDP Statistical Software, PC Dynamic 7.0.

[B21] Bremnes RM, Sundstrom S, Aasebo U, Kaasa S, Hatlevoll R, Aamdal S, Norweigian Lung Cancer Study Group (2003). The value of prognostic factors in small cell lung cancer: results from a randomised multicenter study with minimum 5 year follow-up. Lung Cancer.

[B22] Ando S, Suzuki M, Yamamoto N, Iida T, Kimura H (2004). The prognostic value of both neuron-specific enolase (NSE) and Cyfra21-1 in small cell lung cancer. Anticancer Res.

[B23] Oshita F, Kameda Y, Hamanaka N, Saito H, Yamada K, Noda K, Mitsuda A (2004). High expression of integrin beta1 and p53 is a greater poor prognostic factor than clinical stage in small-cell lung cancer. Am J Clin Oncol.

[B24] Oshita F, Kameda Y, Ikehara M, Tanaka G, Yamada K, Nomura I, Noda K, Shotsu A, Fujita A, Arai H, Ito H, Nakayama H, Mitsuda A (2002). Increased expression of integrin beta1 is a poor prognostic factor in small-cell lung cancer. Anticancer Res.

[B25] Blackhall FH, Pintilie M, Michael M, Leighl N, Feld R, Tsao MS, Shepherd FA (2003). Expression and prognostic significance of kit, protein kinase B, and mitogen-activated protein kinase in patients with small cell lung cancer. Clin Cancer Res.

